# An image and text-based fake news detection with transfer learning

**DOI:** 10.1371/journal.pone.0324394

**Published:** 2025-06-17

**Authors:** Esther Irawati Setiawan, Patrick Sutanto, Christian Nathaniel Purwanto, Joan Santoso, FX Ferdinandus, Nemuel Daniel Pah, Mauridhi Hery Purnomo

**Affiliations:** 1 Information Technology Department, Institut Sains dan Teknologi Terpadu Surabaya, Surabaya, Jawa Timur, Indonesia; 2 Electrical Engineering Department, University of Surabaya, Surabaya, Jawa Timur, Indonesia; 3 School of Engineering, RMIT University, Melbourne, Victoria, Australia; 4 Electrical Engineering Department, Institut Teknologi Sepuluh Nopember, Surabaya, Jawa Timur, Indonesia; Tianjin University, CHINA

## Abstract

Fake news has emerged as a significant problem in today’s information age, threatening the reliability of information sources. Detecting fake news is crucial for maintaining trust and ensuring access to factual information. While deep learning offers solutions, most approaches focus on the text, neglecting the potential of visual information, which may contradict or misrepresent the accompanying text. This research proposes a multimodal classification approach that combines text and images to improve fake news detection, particularly in low-resource settings where labeled data is scarce. We leverage CLIP, a model that understands relationships between images and text, to extract features from both modalities. These features are concatenated and fed into a simple one-layer multi-layer perceptron (MLP) for classification. To enhance data efficiency, we apply LoRA (Low-Rank Adaptation), a parameter-efficient fine-tuning technique, to the CLIP model. We also explore the effects of integrating features from other models. The model achieves an 83% accuracy when using LoRA in classifying whether an image and its accompanying text constitute fake or factual news. These results highlight the potential of multimodal learning and efficient fine-tuning techniques for robust fake news detection, even with limited data.

## Introduction

Fake news, the deliberate fabrication or presentation of misleading information as genuine news, has become a significant problem in today’s information age [1]. The term “fake news” refers to deliberately fabricated or misleading information presented as genuine news. These false narratives can spread rapidly through various media channels, including social media platforms [2], websites, and even traditional news outlets, creating a range of negative consequences.

In a digital world where information is readily available, it can be challenging to distinguish between accurate and false reports. Fake news often mimics the style and format of legitimate journalism, making it difficult for individuals to discern fact from fiction. As a human society, we still haven’t found any clear and total solution to the fake news problem. When people unknowingly consume false information, their beliefs, decisions, and actions may be based on inaccurate or biased data. They will lead to adverse outcomes in both personal and societal contexts.

Fake news can have profound and far-reaching adverse effects on human society. For example, during the COVID-19 pandemic, a surge of misinformation regarding vaccines, including false claims about microchips and other fabricated conspiracies, circulated widely. This kind of fake news led to vaccine hesitancy, worsening the pandemic. The rapid spread of such misinformation on social media, often in images and text, highlights the urgent need for effective detection methods.

Moreover, the sheer volume of fake news content makes manual verification impractical. Social media platforms amplify the spread of misinformation, making it more severe and difficult to control. Given the scale of this issue, automated detection methods that can analyze both text and images are essential for mitigating the harmful impact of fake news. Recently, deep learning methods have shown promising results in fake news detection, as shown in [3–5]. However, relatively few studies have been done on the problem of fake news, even though it’s a very important problem that needs to be solved.

Recent research shows that deep learning is crucial in detecting fake news. Several methods, including BERT [6,7] and Graph Neural Network (GNN) [8], have outperformed results on fake news detection. BERT is one of the best methods for detecting real and fake news, where it can achieve 99.2% accuracy and 99,3% precision on a fake news dataset [3]. Meanwhile, graph neural networks can catch the interactions between the elements in the graph, such as the similarity between document content [4].

Due to their complexity, identifying hoaxes is challenging. Extensive research about this problem employs various methods, such as LSTM [9] (Long short-term Memory) and BiLSTM (Bidirectional Long short-term Memory) [10], and machine learning methods such as Naive Bayes [11], Passive Aggressive Classifier (PAC) [12], and Random Forest. Among these methods, Random Forest can achieve the accuracy of 62.37% [13].

Fake information has become uncontrollable, especially in the era of COVID-19 [14]. Therefore, there is pressure to develop new methods to detect fake news effectively, specifically related to the pandemic. Combining named entity recognition (NER) and stance classification techniques, a new method is developed to capture contextual information and assess the alignment between named entities and claims in news articles [15].

Social media is the best tool for sharing fake news in any situation [16]. Therefore, identifying and labeling social media content is a demanding problem due to the massive amount of heterogeneous content. However, it remains a compelling solution. Its adaptability and learning ability have proven effective in tackling the dynamic landscape of fake news. These approaches have demonstrated an impressive accuracy rate of 85% across different models, emphasizing their efficiency in combating misinformation [17].

As previously mentioned, BERT (Bidirectional Encoder Representations from Transformers) is suitable for capturing fake news. In line with this, researchers propose a BERT-based deep learning approach, FakeBERT [18]. This approach involves the fusion of distinct parallel blocks of a single-layer Convolutional Neural Network (CNN) utilizing various kernel sizes and filters with BERT. This amalgamation effectively addresses the issue of ambiguity in fake news detection. The experimental results demonstrate the superiority of FakeBERT, achieving an impressive accuracy of 98.9%, surpassing the performance of previous models, and establishing its effectiveness in accurately identifying fake news [18].

However, most fake news detection models assume access to abundant labeled data, which is often complex to collect as it needs human annotations. Using low-resource data to train the model often causes an overfitting problem, worsening performance generalization performance. To address this limitation, we investigate using a parameter-efficient fine-tuning problem called LoRA (Low-Rank Adaptation), which freezes the original parameters of the model to avoid overfitting.

Some of the deep learning methods leverage multimodal domains like Vision-Language, as this information is pioneered by the CLIP Method. CLIP is very powerful in zero-shot recognizing images and text [19]. CLIP uses 2 models to encode different modalities (image/text) and is trained such that the image and its text description have a close distance and are far from other image text description [20]. CLIP can easily detect fake news images on social media [5,21].

The image processing part of CLIP uses the ResNet and Visual Transformer (ViT) Method. ResNet learns about residual functions with reference to layer inputs, and these residual networks can be easily optimized and can gain accuracy from increased depth [22]. Meanwhile, ViTs use self-attention mechanisms to capture relationships between different image regions [23]. ViT has gained recognition for its effectiveness in handling the ImageNet dataset, which contains millions of labeled images and thousands of object categories [24]. It has ranged from testing and developing computer vision algorithms to exploring natural language processing and semantic relationships between objects [25].

A hybrid bidirectional LSTM with transformer-based embedding is used for Indonesian news stance classification, capturing sequential language patterns and rich contextual representations to achieve enhanced performance compared to traditional approaches [26]. Noisy text supervision from large-scale web data is leveraged to scale visual and vision-language representation learning, resulting in notable improvements in transfer learning and zero-shot performance on downstream tasks [27].

Transformers [28] is CLIP’s text processing. It has been announced as a powerful and effective approach for sequence-to-sequence modeling in NLP and other domains [28] because it can learn rich representations of language that capture relationships between words and phrases in both directions. These representations can be fine-tuned for a wide range of downstream NLP tasks.

Data augmentation techniques play a crucial role in improving deep learning performance. Traditional, generative, and adversarial approaches are widely used to enhance model robustness [29]. The mixup method generates new samples through convex combinations of existing examples and labels, smoothing decision boundaries and increasing robustness [30]. Randaugment applies uniformly selected transformations with a reduced hyperparameter search space to improve image classification accuracy [31]. Inspired by this, we propose a new method to classify multimodal fake news based on deep learning.

This paper’s main contribution is demonstrating an effective and efficient approach for multimodal fake news detection, particularly under low-resource constraints. We achieve this by leveraging the pre-trained CLIP model to extract joint image-text features and applying LoRA, a parameter-efficient fine-tuning technique. Our key contributions include: (1) showing the significant advantage of combining image and text modalities over unimodal approaches using CLIP; (2) proving the efficacy of LoRA for adapting CLIP to fake news detection with limited data (4000 samples), enhancing performance while mitigating overfitting; and (3) providing empirical evidence that the CLIP+LoRA configuration outperforms linear probing, alternative backbones (ALIGN), and hybrid model combinations in this low-resource setting.

Our work is divided into several sections. Section 1 discusses the introduction and research motivation of this study. Section 2 elaborates on the overall approach to Fake News Classification. Section 3 describes the experiments. Finally, Section 4 presents the conclusion and suggestions for future work.

## Fake news classification

### System architecture

In this work, we explore the potential of combining image and text modalities to classify whether news is fake or factual based on the image and its title. We assume a low-resource setting, as is often the case in real-world applications. To improve fake news classification, we explore various methods with the CLIP model under linear probing settings and investigate using a similar model, ALIGN. Additionally, we consider the application of LoRA (Low-Rank Adaptation) for fine-tuning the CLIP model.

The implementation process begins with collecting data from social media and Indonesian news sources, along with multimodal datasets. Afterward, a data-cleaning process is conducted to ensure quality. Additional features that may enhance classification accuracy are extracted, followed by the formation of word and image vectors using CLIP. A classifier model is then developed to perform stance classification.

Furthermore, we examine the impact of integrating features extracted from different models, such as ResNets, CLIP, and DistilBERT, to assess their contribution to performance improvement. Our findings indicate that combining image and text modalities significantly enhances classification performance. However, integrating features from additional models did not yield improvements, likely due to redundancy. Lastly, we demonstrate that parameter-efficient fine-tuning with LoRA improves performance without overfitting, even in data-scarce scenarios.

**Fig 1:**
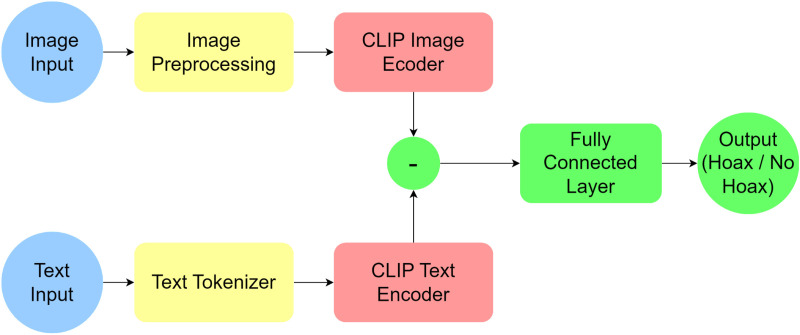
System Architecture.

The algorithm of the system architecture in Fig 1 is Algorithm 1. The input text and image will be put into the CLIP model. Later, 2 outputs will be produced, namely the vision model output and the text model output. These two outputs will be combined using the concat operation to produce an output with a size of 768 + 512 to be forwarded to the fully connected layer to produce a classification of the news given in the input in the form of image and text.

**Algorithm 1 CLIP Model**

01: class ClipCustom(nn.Module):

02:  def _init_(self):

03:   super(ClipCustom,self)._init_()

04:   self.model = CLIPModel.from_pretrained(model_id)

05:   for param in self.model.parameters():

06:    param.requires_grad = False

07:   self.fc = nn.Linear((768+512),1)

08:  def forward(self, inputs):

09:   x = self.model(**inputs)

10:   x = torch.cat((x[‘vision_model_output’][‘pooler_output’], x[‘text_model_output’][‘pooler_output’]), 1)

11:   x = self.fc(x)

12:   return x

CLIP is a model that processes images and text and generates embeddings for each input modality. These embeddings are then concatenated and passed through a fully connected (FC) layer, also known as the classifier head, to predict the corresponding labels. The process involves transforming visual and textual information into a unified representation, allowing for more accurate and comprehensive predictions.

CLIP is initially designed for image captioning tasks by empowering contrastive objectives in the learning phase. This model can provide image and text representation to determine their alignment. As a result, this vanilla version of the CLIP model can only determine if a text describes a corresponding image. In our case, we must decide whether an image and a text are fake or fact. Therefore, we must add another layer to capture fake signals from CLIP representation.

The fake checking task is done after the caption checking task. After getting the image and text input, CLIP will decide if both inputs are aligned. Then, the fully connected layer will decide whether they are fake or fact. We can infer that an unaligned connection between image and text is considered fake news. In contrast, an aligned connection between image and text is not always factual news. The text may describe the image well but might give misleading information.

We are considering using a LoRA adapter to fine-tune the CLIP model and improve performance. LoRA is a parameter-efficient fine-tuning technique that adapts large pre-trained models to specific tasks or domains without modifying most parameters. It’s beneficial when dealing with massive models like large language models (LLMs), where full fine-tuning can be computationally expensive and storage intensive. The core idea is to inject trainable low-rank matrices into the pre-trained model’s layers, keeping the original weights frozen.

Instead of updating the entire weight matrix $W\in R^{d\times k}$ of a layer during fine-tuning, LoRA introduces two smaller matrices: $A\in R^{d\times r}$ and $B\in R^{r\times k}$, where the rank r is much smaller than d and k. The forward pass of the layer is then modified to include the product of these low-rank matrices:


$$h=W_{0}x+\Delta Wx=W_{0}x+BAx$$ (1)

Here, $W_{0}$ represents the original frozen pre-trained weights, and the change in weights ΔW is approximated by the product BA. The A matrix is initialized randomly, and B is initialized at 0 to stabilize training. LoRA then scales ΔW by $\frac{\alpha }{r}$. Only A and B are updated during fine-tuning, while $W_{0}$ remains untouched. This significantly reduces the number of trainable parameters, as r is chosen as a small value (e.g., 4, 8, 16).

### Dataset

**Fig 2:**
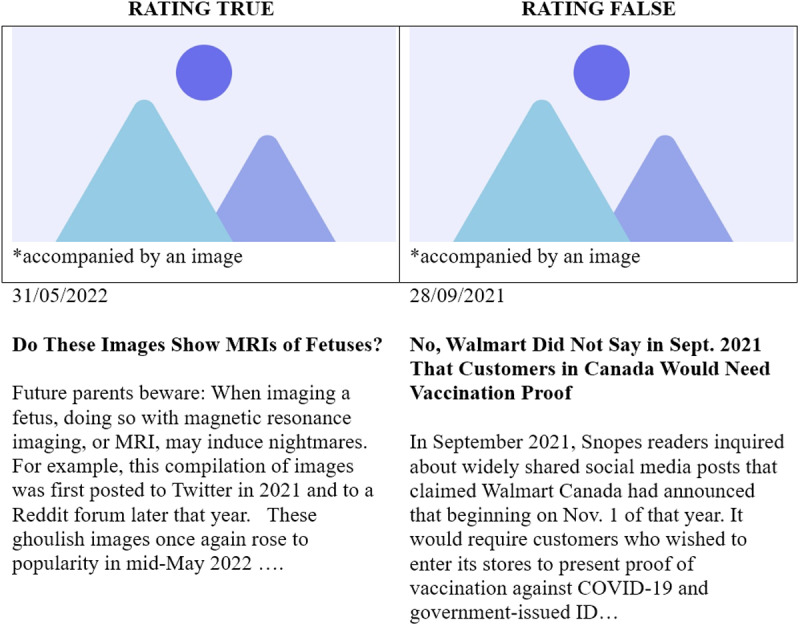
Snopes Dataset example.

This paper uses the multimodal Fakeddit dataset provided by Nakamura, Kai, et al. [32] and the we also crawled from Snopes medical category as our dataset as in [33]. The Fakeddit dataset is a collection of Reddit posts designed for researchers studying fake news. Each post includes a text title, an image, and labels indicating whether the content is real or fake. Unlike other datasets that might focus only on text or have a limited number of posts, Fakeddit provides both text and images from a wide range of topics, reflecting the diversity of discussions on Reddit. This multimodality (Text and images) allows us to develop and test more sophisticated models for detecting fake news, considering visual and textual cues. The crawled data from Snopes is as in Fig 2.

**Fig 3:**
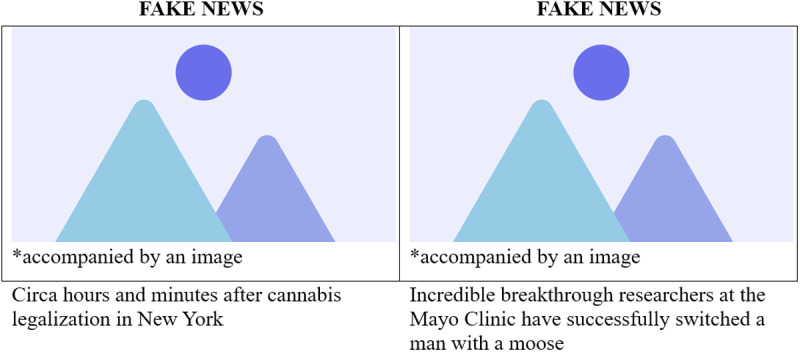
Fakeddit Dataset example.

To investigate the performance of fake news detection models in a low-resource setting, we opted to sample a subset of 4,000 data points from the expansive Fakeddit dataset as shown on Fig 3. This approach aims to simulate scenarios where labeled data is scarce, a common challenge in many real-world applications, particularly in the rapidly evolving landscape of online misinformation. By working with this reduced dataset, we aim to understand how well models can generalize and accurately classify fake news when trained on limited examples.

Furthermore, our study with the 4,000-sample subset will provide a benchmark for future low-resource fake news detection research. We intend to thoroughly analyze the performance of various models on this subset, comparing their accuracy, precision, and recall understanding the trade-offs involved in training with limited data. Insights gained from this study will contribute to developing more efficient and practical solutions for combating the spread of fake news, even in resource-constrained environments.

### Formation of word and image vector

We use deep learning methods that leverage multimodal domains, like Vision-Language, pioneered by the CLIP method [5]. CLIP is very powerful in zero-shot recognition of images and text. Because of its capability to perform many tasks, we hypothesized that we could use this method to solve the image—and text-based fake news problem.

CLIP uses two models to encode different modalities (image/text) and is trained so that the image and its text description are close to each other and far from other image text descriptions.

The image processing part of CLIP [5] uses ResNet [22,34] or Visual Transformer (ViT) [23], which takes image input that has been preprocessed using z-norm with mean and standard deviation estimated from the ImageNet dataset [25]. The output of the image encoder is a vector. If using ViT, we can use [CLS] token representation; using a residual network, we can use the pooled output.

The text processing part of CLIP uses a Transformer that takes tokenized text and outputs a vector, which can also be obtained from the [CLS] token. The output of text and image can be aggregated using a concatenation operator. The result of concatenated text and image vectors will be the data that will train the model.

### Classifier

After getting the features that will be processed, making and training the model can be completed. Multi-Layer Perceptron (MLP) will be used as the classifier of this model. The output will be processed further using nonlinearity and trained with specific loss for downstream tasks. In this work, we use a sigmoid function for the nonlinearity function and train the network using binary-cross entropy loss and end-to-end.

For simplicity, we freeze the CLIP model parameter and only train the MLP part, where the MLP used is a one layer network. Due to resource constraints, we use a straightforward method for training and evaluating the model. We trained the MLP parameter and optimized using Adam with a 2e-4 learning rate. We train the model with 50 iterations and a mini-batch size of 8; this training method will allow fast training even only using a low amount of resources.

### Performance evaluation

Performance evaluation is based on model prediction accuracy compared to the true label.


$$\mathrm{\backslash [}Accuracy=\frac{TP+TN}{TP+FP+TN+FN}\mathrm{\backslash ]}$$ (2)

Accuracy is the difference between correct predictions (true or false because there are only 2 labels) and all of the predictions the model makes.

### Experiments

Our first evaluation experiment uses the Fakeddit dataset. We trained the model using 8000 random images and caption text and evaluated the model trained using 3502 random images and caption text.

In this experiment, we don’t fine-tune the model. Instead, we freeze the CLIP layer and only train the MLP layer. We train the model using a learning rate of 2e-4 and a batch size of 8.

Table 1 shows that using text + image as input achieves more accuracy than using only text or image. If we use Multimodal data, we achieve an accuracy of 81.32%. However, if we only use Text or Images as the data for the training and testing, we can only achieve 76.9% when using only text and 75.41% when using only images.

**Table 1:** Fakeddit Dataset Experiment.

Input	Accuracy
**Text Only**	0.769
**Text + Image**	0.813
**Image Only**	0.754

We also perform a comparison using another model besides CLIP. For this experiment, we choose to use the ALIGN model. The results are shown in Table 3. ALIGN (A Large-scale ImaGe and Noisy-text embedding) is a deep learning model similar to CLIP, developed by Google AI, that learns joint representations of images and text from a massive dataset of noisy image-text pairs obtained from the web. It uses a dual-encoder architecture with a contrastive loss to align the visual and textual representations in a shared embedding space. What distinguishes ALIGN is its focus on training efficiency and scalability, allowing it to be trained on a dataset of over one billion image-text pairs, significantly larger than CLIP’s dataset. While it sacrifices some level of curation for sheer scale, ALIGN demonstrates that training on massive amounts of noisy data can still produce powerful and robust visual-textual representations suitable for tasks like cross-modal retrieval and zero-shot image classification.

For this experiment, instead of using 8000 data to train and evaluate, we used 4000 data. We show that even when we use half of the data, the model’s performance did not degrade. This is because we freeze the CLIP layer and only train the layer above CLIP, which enabled the method to obtain good performance even when using a smaller amount of data. We also show that using the CLIP model performs better than the ALIGN model.

We also use data from the Fakeddit dataset to train and predict the data from the Snopes dataset. However, we found that text significantly influences the prediction of whether the news is fake on the Snopes dataset. The result is shown in the Table 2.

**Table 2:** Align Model Comparison.

Model	Accuracy
**ALIGN**	0.771
**CLIP**	0.815

**Table 3:** Fakeddit + Snopes Dataset Experiment.

Input	Accuracy
**Text Only**	0.714
**Text + Image**	0.536
**Image Only**	0.438

Table 3 shows that text + image as input achieves less accuracy than when we use only text, however it shows better results than only using an image as the input. When we use text only as input, we can achieve 71.38% accuracy, but only 53.61% and 43.75% accuracy when using multimodal text + image and only image.

Our approach is to classify fake news using combined images and text features. First, we consider the linear probing approach to show the importance of using image and text features. We embed the image and title text using CLIP image and text encoder. Then, we concatenate the pooled representation and feed it to the linear layer to predict whether the article is fake. We also consider a similar approach but use only image/text modality alone without concatenating. We first investigate the setting where the parameters of the CLIP model are frozen, which made the method very efficient to train. Moreover, even when the data is low-resource, as the model parameters are not updated, we can avoid overfitting problems. Our experiment uses the subset of the Fakeddit dataset. We use 4000 random images and caption text to train the model and 4000 random images and caption text to evaluate the model trained using the 4000 data.

Table 4 shows that text + image as input achieves more accuracy than when we use only text or image. If we use Multimodal data, we achieve an accuracy of 81.4%. However, if we only use Text or Images as the data for the training and testing, we can only achieve 76.1% when using only text and 74.5% when using only images. This shows that using both image and text features significantly outperforms their unimodal counterparts. Next, we investigate whether another model can be used and how it compares with the CLIP model. To investigate this, we consider using the ALIGN model to replace the clip model.

**Table 4:** Fakeddit Dataset Experiment.

	CLIP Model Image + text	CLIP Model Image	CLIP Model Text
**precision**	**0,821**	0,748	0,736
**recall**	**0,742**	0,638	0,716
**f1_score**	**0.779**	0.689	0.726
**accuracy**	**0.814**	0.745	0.761

As displayed in Table 5, CLIP outperforms ALIGN. We think this is because the ALIGN model representation is not as good as CLIP’s, even though when ALIGN is used for retrieval or zero-shot, it can achieve similar performance compared to CLIP. Next, we investigate the hybrid approach of combining the CLIP model with representation from other ResNet models and distilleries. The pseudocode is shown on Algorithm 2.

**Table 5:** Detailed Comparison Using ALIGN.

	CLIP Model Image + text	ALIGN Model Image + text
**precision**	**0,821**	0,764
**recall**	**0,742**	0,697
**f1_score**	**0.779**	0.729
**accuracy**	**0.814**	0.771

**Algorithm 2 Mixed Model**

01:x_mm = self.multimodal_model(**inputs_multimodal)

02:x_mm = torch.cat((x_mm[‘vision_model_output’][‘pooler_output’], x_mm[‘text_model_output’][‘pooler_output’]), 1)

03:x_mm = self.multimodal_fc(x_mm)

04:

05:x_v = self.vision_model(**inputs_image, output_hidden_states = True)

06:x_v = F.avg_pool2d(x_v[‘hidden_states’][-1], 7, 7)

07:x_v = x_v.reshape(len(x_v), x_v.shape[1])

08:x_v = self.vision_fc(x_v)

00:

10:x_t = self.text_model(**inputs_text)[‘last_hidden_state’]

11:x_t = x_t[:,0]

12:x_t = self.text_fc(x_t)

13:

14:x_all = torch.cat((x_mm, x_v, x_t), 1)

15:output = self.output_linear(x_all)

16:

We concatenated the embedding of CLIP as before and fed it with a linear layer that maps it into a 768-dimensional dimension. We also fed both the Resnet feature and the distillery feature with a linear layer and obtained 384 features each. We then concatenated all the features to obtain 1536 features, which we fed to the final linear head to classify whether it was fake news or not. The result is shown on Table 6.

**Table 6:** Effect of LoRA Fine-Tuning on CLIP Model Performance with Varying Ranks.

	CLIP Model Image + Text	All Hybrid
**precision**	0,821	0,820
**recall**	0,742	0,722
**f1_score**	0.779	0.768
**accuracy**	0.814	0.807

We observe that using hybrid models is not effective. Their use of a hybrid model did not result in significant improvement compared to using a CLIP model only, and it added additional time and complexity. We think that this is caused by the feature’s redundancy, even if we use 2 different models with different architectures. Lastly, we consider LoRA parameter-efficient fine-tuning to improve performance further. Next, we consider the application of LoRA to fine-tune the models.

We show on Table 7 that using LoRA can significantly improve performance, albeit with additional training costs. We also show that training more rank results in performance improvements. Moreover, we did not see any hint of overfitting, which shows the effectiveness of using LoRA under low-resource settings. We can run all the experiments on free-tier Google Colab, which shows the efficiency of our method. The model can even be trained without GPU, albeit longer.

**Table 7:** Effect of LoRA Fine-Tuning on CLIP Model Performance with Varying Ranks.

	CLIP Model Image + Text	CLIP LoRA r = 4	CLIP LoRA r = 8	CLIP LoRA r = 16
**precision**	**0,821**	0,880	0,881	0,875
**recall**	0,742	0,693	**0,694**	0,727
**f1_score**	0.779	0.775	0.776	**0.794**
**accuracy**	0.814	0.823	0.823	**0.833**

## Conclusion

This research demonstrates the effectiveness of a multimodal approach, combining image and text features, for detecting fake news, particularly in low-resource settings. By leveraging the pre-trained CLIP model, we were able to extract meaningful representations from both modalities, which, when concatenated and fed into a simple multi-layer perceptron, achieved an F1 score of 86.02% using a LoRA rank of 16 on a subset of the Fakeddit dataset. This significantly outperformed models using only image or text features, highlighting the importance of considering both modalities for accurate classification.

Our experiments also revealed that incorporating features from other models, such as ResNet and DistilBERT, did not yield significant improvements, suggesting potential redundancy in the extracted features. Notably, applying LoRA, a parameter-efficient fine-tuning technique, proved crucial for enhancing performance without overfitting, even with limited training data. This underscores the potential of LoRA for adapting large pre-trained models to specific tasks in data-scarce scenarios.

Furthermore, our findings indicate that multimodal data—including images and text—consistently outperforms models relying on a single modality. This approach improves fake news classification and shows promise in filtering datasets and identifying out-of-domain images, making it useful for training models on more relevant data.

The ability to train our models efficiently, even without a GPU, demonstrates the practicality of our approach for real-world applications. While LoRA fine-tuning does introduce a slight increase in training time, the significant performance boost justifies its use.

In conclusion, our multimodal approach, combined with efficient fine-tuning using LoRA, offers a promising solution for robust fake news detection, especially when labeled data is scarce. This research contributes to developing more effective and accessible tools for combating misinformation in the digital age. Future work could explore the integration of other modalities, such as user comments or network information, to further enhance detection accuracy and refine dataset selection. Additionally, expanding the dataset could improve model generalization and improve classification performance.
